# Electrically tunable two-dimensional heterojunctions for miniaturized near-infrared spectrometers

**DOI:** 10.1038/s41467-022-32306-z

**Published:** 2022-08-08

**Authors:** Wenjie Deng, Zilong Zheng, Jingzhen Li, Rongkun Zhou, Xiaoqing Chen, Dehui Zhang, Yue Lu, Chongwu Wang, Congya You, Songyu Li, Ling Sun, Yi Wu, Xuhong Li, Boxing An, Zheng Liu, Qi jie Wang, Xiangfeng Duan, Yongzhe Zhang

**Affiliations:** 1 Key Laboratory of Optoelectronics Technology, Ministry of Education, Faculty of Information Technology, Beijing University of Technology, Beijing, 100124 China; 2 Key Laboratory of Advanced Functional Materials, Ministry of Education, Faculty of Materials and Manufacturing, Beijing University of Technology, Beijing, 100124 China; 3 Centre for OptoElectronics and Biophotonics, School of Electrical and Electronic Engineering, Nanyang Technological University, Singapore, 639798 Singapore; 4 Department of Chemistry and Biochemistry, University of California, Los Angeles, Los Angeles, CA 90095 USA; 5 California NanoSystems Institute, University of California, Los Angeles, Los Angeles, CA 90095 USA; 6 Beijing Key Laboratory of Microstructure and Properties of Solids, Faculty of Materials and Manufacturing, Beijing University of Technology, Beijing, 100124 China; 7 School of Materials Science and Engineering, Nanyang Technological University, Singapore, 639798 Singapore; 8 Centre for Disruptive Photonic Technologies, School of Physical and Mathematical Sciences, Nanyang Technological University, Singapore, 637371 Singapore

**Keywords:** Sensors, Two-dimensional materials

## Abstract

Miniaturized spectrometers are of considerable interest for their portability. Most designs to date employ a photodetector array with distinct spectral responses or require elaborated integration of micro & nano optic modules, typically with a centimeter-scale footprint. Here, we report a design of a micron-sized near-infrared ultra-miniaturized spectrometer based on two-dimensional van der Waals heterostructure (2D-vdWH). By introducing heavy metal atoms with delocalized electronic orbitals between 2D-vdWHs, we greatly enhance the interlayer coupling and realize electrically tunable infrared photoresponse (1.15 to 1.47 μm). Combining the gate-tunable photoresponse and regression algorithm, we achieve spectral reconstruction and spectral imaging in a device with an active footprint < 10 μm. Considering the ultra-small footprint and simple fabrication process, the 2D-vdWHs with designable bandgap energy and enhanced photoresponse offer an attractive solution for on-chip infrared spectroscopy.

## Abstract (web summary)

Miniaturized infrared spectrometers are required for imaging and remote sensing applications, but they are usually characterized by a cm-scale footprint. Here, the authors report the realization of near-infrared spectrometers based on Au-atom-intercalated ReS_2_/WSe_2_ heterostructures with an active footprint < 10 μm and electrically tunable photoresponse.

## Introduction

Spectrometers are essential instruments in modern scientific research and engineering production, for instance, geological prospecting, medical examination, spectral imaging, and remote sensing. Most spectrometers are bulky and expensive due to their complex mechanical parts, including motorized optical gratings and interferometers^1,2^. Miniaturized spectrometers with a significant cost and footprint reduction are attractive for portable analytic tools, smart wearable devices etc^3–5^. There are two typical underlying strategies for compact spectrometers. One approach is to integrate photodetectors with three types of separate optical elements^6–12^, including spatially dispersive grating, tunable filter arrays, and Fourier transform interferometers^13–17^. It has been shown that this strategy provides excellent performance but is difficult to scale below the sub-millimeter scale due to the negative impact induced by fundamental physical limitations in optical path length. The other approach is to employ multifunctional photodetector arrays such as nanowire with spatial compositional gradients^18^ and structural vertical silicon nanowire arrays^19^, with special computational reconstructive algorithms. These reconstructive spectrometers require not optical components and can be readily scale down to sub-millimeter footprints. However, infrared (IR) band operation, precision manufacturing complexity, as well as the trade-off between footprint and number of photodetectors remains challenging.

The van der Waals heterojunctions (vdWH) formed between distinct 2D transition metal dichalcogenides (TMDs) with type II energy band structure alignment provides a versatile platform for exploring interlayer excitons^20–23^. With the conduction band minimum (CBM) and valence band maximum (VBM) of the heterojunction localize in different layers, such typical type II heterojunctions opens a tunable degree of freedom to engineering interlayer optical transition in the IR regime beyond the limit of the intrinsic optical band gap of the constituent material^24^. Indeed, direct observation of interlayer optical excitation (IEX) infrared photoresponse was reported^25^. Meanwhile, with the strong electrically reconfigurable feature^26–31^, the type-II 2D-vdWH offer an intriguing system for electrically tunable infrared photoresponse. Unfortunately, the transition dipole moment of the interlayer exciton, as well as oscillator strength of IEX is usually too weak in 2D-vdWH to achieve effective photodetection and significant photoresponse, because of the spatial separation between electron wavefunctions (at CBM) and hole wavefunctions (at VBM) in different layers^32^.

Herein we report a generic method to enhance the interlayer transition dipole moment of IEX in 2D-vdWH, by intercalating heavy metal atoms (e.g., Au atoms) at the interface of 2D-vdWH (ReS_2_/Au/WSe_2_). Our first-principles calculations indicated that the transition dipole moment of the interlayer exciton is enhanced significantly due to the delocalized orbitals of Au atom bridging the heterobilayers, which is confirmed by the strong photoresponsivity with roughly two-times increasing. Furthermore, we show ReS_2_/Au/WSe_2_ exhibit a unique gate-tunable near-infrared (NIR) photoresponse. Exploiting the gate-tunable photoresponse and the ridge regression algorithm, we realize an ultra-miniaturized NIR spectrometer and spectral imager with a footprint of 6-microns.

## Results

### Device scheme and theoretical study

**Fig. 1:**
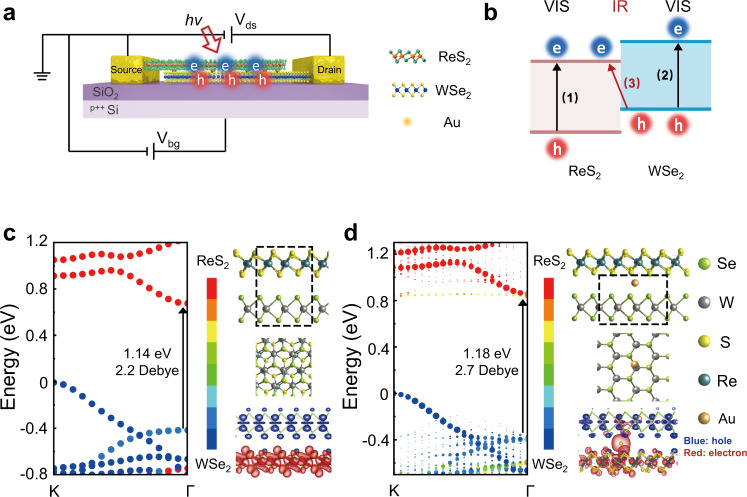
Design of 2D van der Waals heterojunction (vdWH) spectrometer. **a** Schematic drawing of the 2D-vdWH spectrometer. The heterojunction is intercalated by heavy metal Au atoms to construct ReS_2_/Au/WSe_2_, where the junction would promote the separation of photo-excited electrons and holes. V_ds_ and V_bg_ are bias voltage and back gate voltage, respectively. ‘*hv*’ and the red arrow represent the incident light. **b** Photo-excited transition path in the heterojunction, including intralayer transition (1) & (2) and interlayer transition (3), where the path (3) corresponds to lower transition energy to near-infrared (IR) band than both band gaps of ReS_2_ and WSe_2_ in visible (VIS) band. Atomic geometries, band structures, and electron distribution of conduction band minimum in WSe_2_ layer (and hole distribution of valance band maximum in ReS_2_ layer) for both **c** pristine ReS_2_/WSe_2_ and **d** ReS_2_/Au/WSe_2_ with Au intercalation heterojunctions, respectively. In the optimized geometry, intervals of S-Au and Se-Au are 2.47 and 2.56 Å, respectively. The lighter dots in **d** representing the additional few densities of states in k-space by the changed lattices of 2D-vdWH due to the participation of Au atom. The black arrows indicate the transition energy at Г point for the two heterojunctions. The top-view atomic arrangements correspond to the region marked by black dash line in the side-view atomic arrangement. The electron density distribution shows that the electron wavefunctions in the ReS_2_ layer are delocalized and partially delivered to the WSe_2_ layer through Au atoms. Red and blue represent the relative contribution of the states from electrons in ReS_2_ and holes in WSe_2_, respectively.

The schematic diagram of the 2D-vdWH spectrometer and its electrical contact is shown in Fig. 1a. Figure 1b shows a typical Type II band energy of the designed 2D-vdWH, depicting two types of transition channels with (1, 2) intralayer transition and (3) interlayer transition, where interlayer exciton holds low energy to IR band than that of the intralayer exciton. Therefore, the heterojunction of WSe_2_/ReS_2_ could be applied as the NIR materials since their IEX energy is less than 1.2 eV^33^. Furthermore, by coupling with a back-gate electrode to tune the relative band alignment between the top and bottom-layer, the NIR interlayer transition can be further tuned by the vertical gate field, which is essential for constructing a single device spectrometer by using regression algorithm.

However, the spatial separation between electrons in CBM and holes in VBM leads to a very small overlap between their wavefunctions, resulting in the weak transition dipole moment <*Ψ*_*1*_|*M*|*Ψ*_*2*_>. Hence, the interlayer oscillator strength is approximately at least 2 orders of magnitude smaller than that of intralayer transition^24,25^, and it is thus difficult to subsequently measure the gate-voltage tunable spectral photocurrents. More overlaps between electron wavefunction and hole wavefunction could enhance the transition dipole moment as well, which may be achieved by reducing the interlayer distance^34,35^ by using hydrostatic pressure in a diamond anvil cell^36^, which is not easy to maintain for practical applications. Alternatively, intercalating heteroatoms with delocalize atomic orbitals^37^ may offer another approach to enhance the interlayer coupling to achieve higher transition dipole moment of IEX. To explore this concept, we introduce heavy metal Au atoms between 2D heterobilayers to form a ReS_2_/Au/WSe_2_ sandwich structure, in which Au atoms with a large atomic radius and delocalized electronic orbital could enhance wavefunction overlaps between electrons (in CBM) and holes (in VBM), respectively.

We have first conducted first-principles simulations of two modeling structures, (i) ReS_2_/Au/WSe_2_ with Au intercalation, and (ii) pristine ReS_2_/WSe_2_ as a control, to determine if the former one can enhance the infrared absorption of the interlayer exciton in 2D-vdWH. The electronic band structure calculations, including transition dipole moment elements and partial charge distributions, were performed using density functional theory (DFT). The band structure of pristine 2D ReS_2_/WSe_2_ heterostructure presents a standard type-II band alignment, where the CBM is localized on the ReS_2_ layer, and the VBM is mainly contributed by the WSe_2_ layer (Fig. 1c), respectively. The indirect bandgap (Γ to K) and the direct bandgap (Γ to Γ) were obtained as 0.7 eV and 1.14 eV in theory. Meanwhile, we also confirm the theoretical exciton energy of monolayer (ML) WSe_2_ (1.6 eV) and ReS_2_ (1.5 eV) (see Supplementary Fig. 1). The direct interlayer charge transfer exciton should be weak at the ReS_2_/WSe_2_ interface since the VBM and CBM are separated and localized at different layers. The indirect ReS_2_/WSe_2_ bandgap transition also has to satisfy the momentum conservation with the phonon-assisted exciton hopping, which lowers the absorption of interlayer ReS_2_/WSe_2_ exciton. Therefore, such indirect transition is also expected to be weak.

We then focus on enhancing the absorption of direct bandgap, which is still based on the interlayer transition in infrared spectroscopy (1.14 eV). The d-orbital of heavy metal Au atom has a strong non-locality effect, which could assist overlap between electron and hole of charge transfer exciton and enhance the ReS_2_/WSe_2_ interlayer transition. Following the Au intercalation (ReS_2_/Au/WSe_2_), we obtained an energy-stable structure by employing the DFT method (Fig. 1d). The intercalation of Au atom is right in the center of the hexahedral cavity consisting of three S of ReS_2_ and three Se of WSe_2_, The strength of interlayer charge transfer exciton from the VBM (in WSe_2_) to the CBM (in ReS_2_) at the Γ point can be derived by the transition dipole moment (${M}_{{{{{{\rm{Z}}}}}}}$) as following equation^38^

1 $${M}_{{{{{{\rm{Z}}}}}}}=\left\langle {\psi }_{{in}}\left|{\hat{r}}_{{{{{{\rm{Z}}}}}}}\right|{\psi }_{{fi}}\right\rangle$$

Where $|{\psi }_{in}\rangle$ and $|{\psi }_{fi}\rangle$ are the wavefunction of the initial and final states, respectively; and ${\hat{r}}_{{{{{{\rm{Z}}}}}}}$ is the position operator along the direction, which is perpendicular to the ReS_2_/WSe_2_ interfacial surface (named as Z direction). With the assistance by the delocalized electronic orbital of Au atom, the ${M}_{{{{{{\rm{Z}}}}}}}$ (between VBM and CBM) increases from 2.2 Debye up to 2.7 Debye. More importantly, the Au atom significantly promotes the wavefunction overlapping between holes and electrons (Fig. 1d). If we consider the absorption is proportional to the square of transition dipole moment, the absorption can be enhanced by ~1.5 times. Similar delocalization is observed in the twisted heterojunction considering the randomness of spatial alignment between ReS_2_ and WSe_2_ (See Supplementary Fig. 2 for detail).

**Fig. 2:**
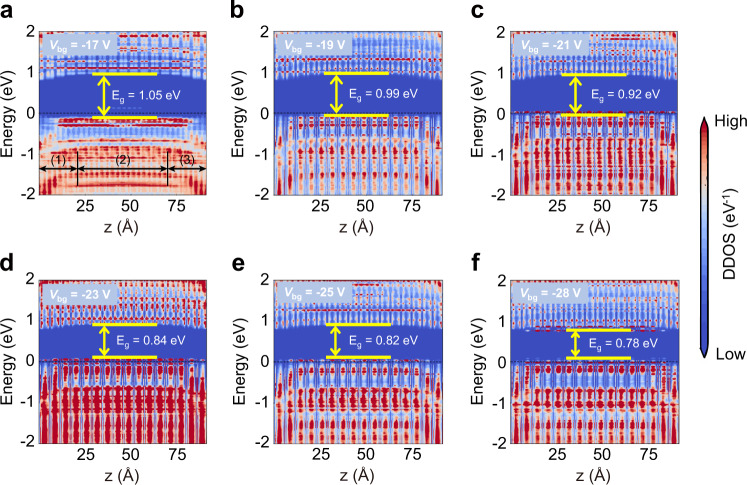
Theoretical study of tunable device density of states (DDOS). **a**–**f** Theoretical calculation of the V_bg_ dependence of device density of states through device simulation. The Fermi level is represented by a black dash line. The color scale is shown on the right of the plot, representing zero-bias device density of states of the monolayer (ML) WSe_2_-ML ReS_2_ transistors. Region (1), (2), and (3) represent left electrode, channel, and right electrode, respectively. E_g_ represents the bandgap of the device channel.

The electrically tunable photoresponse is of great importance to realize the function of a miniaturized spectrometer. Due to the excellent gate controllability related to their atomic thickness, 2D semiconductors usually are convenient to tune the bandgap energy by applying an external gate voltage to control the electronic state distributions of the unfilled conduction band^39^. To confirm that the gate voltage can better control the WSe_2_/ReS_2_ bandgap by redistributing the electronic states in the conduction band, we apply a gate voltage to the ML WSe_2_-ML ReS_2_ transistor (See Supplementary Fig. 3). We predict the device’s density of states (DDOS) with ab initio electronic structure calculations and quantum transport simulations. DDOS is a direct way to reflect the real space energy band distribution in transistors. The bandgaps E_g_ of the transistor are determined from the energy difference between the CBM and VBM. The calculated bandgaps are 1.05, 0.99, 0.92, 0.84, 0.82, and 0.78 eV with the gate voltage at −17, −19, −21, −23, −25, and −28 V, respectively (Fig. 2).

### Optical properties characterization

**Fig. 3:**
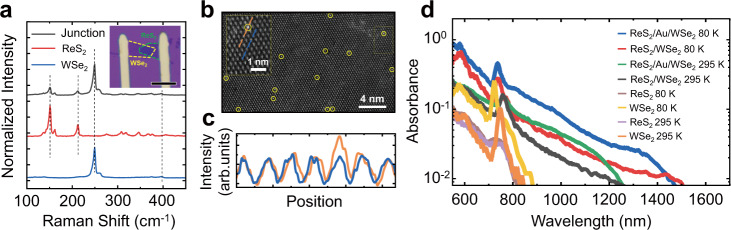
Characterization of Au atom intercalated 2D-vdWH. **a** Raman spectra for ReS_2_ layer, WSe_2_ layer, and ReS_2_/Au/WSe_2_ heterojunction, where the spectrum in the heterojunction region includes all the characteristic peaks from ReS_2_ and WSe_2_, of which the peaks are marked by the vertical black dash lines. Inset: Optical image of ReS_2_/Au/WSe_2_ heterostructure. Yellow and green dashed line depict WSe_2_ and ReS_2_, respectively. The scale bar is 10 μm. **b** HADDF-TEM images for atoms arrangements. The observed metal atoms are circled by the yellow ring. The crystal structure is aligned with the schematic atomic arrangement of WSe_2_. Inset: Zoom-in part of the dash frame. **c** Compared with atom intensities along the orange and blue lined atomic arrangements in the inset of **b**, obvious enhancement of a stochastic atom intensity can be observed, as the yellow circular mark. That illustrates that the Au atom locates right on the top of the wolfram (W) atom at its energy-favorable configuration. **d** The absorption spectra of ReS_2_/Au/WSe_2_, ReS_2_/WSe_2_, ReS_2_, and WSe_2_ at room temperature and 80 K in the visible and near-infrared range.

The Au intercalated 2D-vdWH was fabricated to further achieve experimental demonstration (See method for fabrication detail). The Raman spectra the ReS_2_/Au/WSe_2_ sample indicates that the 2D-vdWH are constructed successfully (Fig. 3a)^40,41^, while the lattice vibration modes of both materials are barely affected by the sandwiched Au. The thickness of ReS_2_ and WSe_2_ are MLs (see Supplementary Figure 4). Meanwhile, energy dispersive X-ray spectroscope (EDS) mapping illustrates the existence of Au (Supplementary Fig. 5)^42^. Furthermore, the high-angle annular dark field transmission electron microscope (HADDF-TEM) image clearly resolves the Au atom right on the top of wolfram (W) atom (Fig. 3b, c) at its energy favorable configuration, which is consistent with the theoretical calculation (Fig. 1d). To confirm that Au atoms enhance the IEX photoresponse in the heterojunction, we have investigated the absorption spectra of ReS_2_/Au/WSe_2_, ReS_2_/WSe_2_, ReS_2_, and WSe_2_ at room temperature and 80 K (Fig. 3d). Compared with the single MLs with a cutoff wavelength below 900 nm, the heterojunctions show a broader absorption in the near-infrared range. Notably, the heterojunction with Au exhibits a clearly enhanced absorption (by nearly 2 times) in the infrared regime, which is consistent with the theoretical calculation results.

### Photoresponse characterization

**Fig. 4:**
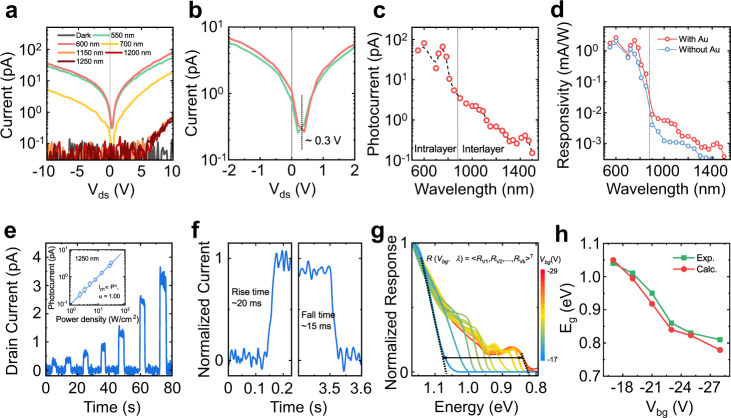
Infrared Photoresponse of the 2D heterojunction by interlayer optical excitation. **a** I-V_ds_ curves of ReS_2_/Au/WSe_2_ at different excitation wavelengths of 550, 600, 700, 1150, 1200, and 1250 nm. **b** Zoom-in part of I-V_ds_ curves in (**a**), where the open circuit voltage of about 0.3 V is marked by the vertical black dash line. **c** Spectral photocurrents of the device from visible to NIR range. When the incident wavelength is less than ~880 nm (marked by the vertical black dashed line), the response is from intralayer transition. On the contrary, it is from interlayer transition. **d** Comparison of spectral responsivities of ReS_2_/Au/WSe_2_ and ReS_2_/WSe_2_, where obvious photoresponse enhancement is observed in the interlayer transition region. **e** Time dependence of photoresponse under 1250 nm illumination, corresponding to the interlayer transition pathway with power intensities from 2 to 40 W/cm^2^. Inset: Power dependence of photocurrents under 1250-nm illumination. The values are fitted by the power law I_ph_ ~ P^α^, where the exponent α = 1.00. **f** One circle on-off switching of photoresponse identifies the response time of this device, demonstrating the fast rise and fall times of ~20 and 15 ms, respectively. **g** Calibrated spectral photoresponse modulated by different V_bg_ experimentally. With the increase of negative V_bg_ from −17 V to −29 V, the cut-off wavelength varies from ~1150 to ~1470 nm, corresponding to the range between 1.07 and 0.84 eV, which also determines the operational spectral range of this spectrometer. The colorbar represents the varied V_bg_. **h** Bandgaps E_g_ for the experimental (Exp.) and calculational (Calc.) ML WSe_2_-ML ReS_2_ transistors against the gate voltage.

We have further evaluated I-V curves of ReS_2_/Au/WSe_2_ device excitated by different wavelength of light (Fig. 4a, b). When VIS light irradiates, the device shows a photovoltaic effect with a large open-circuit voltage of 0.3 V, which proves effective charge transfer and strong coupling of the heterojunction usually appearing in a well-fabricated type II heterostructure^43^. Moreover, the extra infrared photoresponse is observed. Figure 4c shows the measured spectral photocurrents, where the photoresponse in NIR range is deduced from the interlayer transition according to the absorption spectrum. Photoresponsivity comparison between pristine ReS_2_/WSe_2_ heterojunction and Au intercalation ReS_2_/Au/WSe_2_ heterojunction is shown in Fig. 4d during the full photoresponse range. Overall, we see a roughly two-time higher photoresponse in ReS_2_/Au/WSe_2_ heterojunction when compared that in ReS_2_/WSe_2_ within the IEX photoresponse range. The time and power dependences of IEX photoresponse (Fig. 4e, f) illustrate that this 2D-vdWH device exhibits fast response time (around 20 ms) and a linear relationship of photocurrent versus light intensity (slope extremely close to 1). The fast temporal response and the well-defined relationship is essential for the realization of spectral reconstruction, especially in the scanning imaging by the designed device. We then investigate the relationship between V_bg_ and spectral photoresponse. Because the calibration of responsivities R as a function of wavelength and V_bg_ is the precondition to realize the function of spectrometer^18,44^. Finally, the responsivity matrix *R* (*V*_*bgk*_*, λ*) related to wavelength and gate voltage is obtained. With the increase of negative *V*_*bg*_ from −17 V to −29 V, the cut-off wavelengths are from 1150 nm (1.07 eV) to 1470 nm (0.84 eV) (Fig. 4g, h). Interestingly, the experimentally measured bandgaps reduce 0.23 eV, which is in good agreement with our theoretical prediction (0.27 eV, Fig. 2). The variable spectral photoresponse range is the key point to realize this type of reconstructive spectrometer. Therefore, the operation range for this device could be determined as ~1150 to 1470 nm, where the spectral photoresponse of the device could be well tuned by varying *V*_*bg*_ from −17 to −29 V. More details for the principle of this spectrometer are provided in Method. In addition, more devices by randomly stacking the constituent layers with no control on their orientation are fabricated and characterized to demonstrate the reproducible mechanism (Supplementary Figs. 6 and 7).

### Spectroscopy and imaging demonstration

**Fig. 5:**
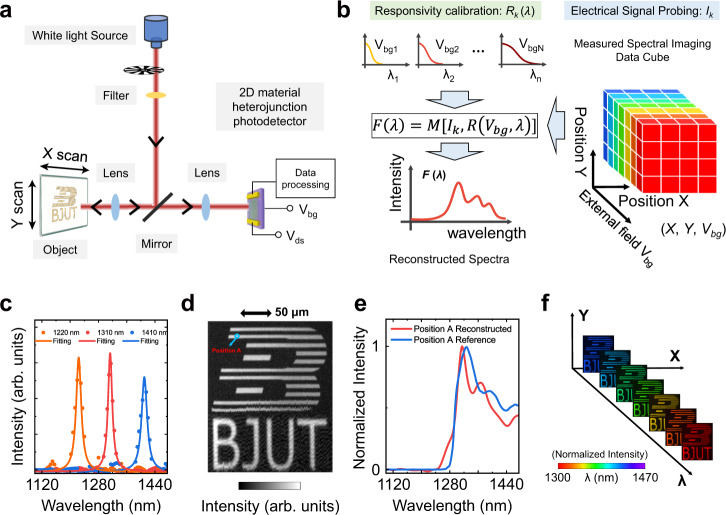
Demonstration of spectrometer and scanning spectral imaging. **a** Schematic diagram of the spectral imaging system. Black arrows indicate the light propagation direction. **b** Principle of the 2D heterojunction-based spectrometer. Especially, for spectral imaging, spectral data cubes consist of location information, and corresponding spectral information at every location point is reconstructed by the V_bg_ modulated photocurrents *I*_k_ according to the relationship between unknown incident light *F*(*λ*), photocurrent *I*_*k*_ and Responsivity *R*. *M* represents unknown non-linear function. **c** Reconstructed spectra from three narrowband spectra of which the peak locations are 1220 nm, 1310 nm, and 1410 nm, respectively. **d** Single-pixel scanning image for the logo of “Beijing University of Technology”. The Scale bar is 50 μm. Broadband white light modulated by an optical filter was applied for illumination to make sure that spectra of incident light is in the operational range of this designed spectrometer. **e** Spectral information of position A in **d**, which is matched well with a result from conventional equipment (blue-line). **f** Images of the logo at some selected wavelength from 1300 nm to 1470 nm are schematically shown. To clearly show the spatial information, false colors are defined for the above spectral range, and the intensities are normalized.

With the calibration of *R* (*V*_*bgk*_*, λ*), the function of a spectrometer and the spectral imager are demonstrated with this designed 2D-vdWH. Figure 5a shows a schematic diagram of the active spectral imaging system and its operational principle. To reconstruct an unknown incident spectrum, there are two steps as shown in Fig. 5b. Firstly, photocurrents matrix ${{{{{\bf{I}}}}}}=\left\langle {I}_{1},{I}_{2},\ldots,{I}_{k}\right\rangle$ at different *V*_*bg*_ is probing under the reflected spectra by the object. Secondly, spectra are reconstructed by the regression algorithm based on the calibrated *R* (*V*_*bgk*_, *λ*). In addition, both the spatial and *V*_*bg*_ dependences of photocurrents need to be recorded to complete spectral imaging, i. e., the data cube. The crucial formula follows,

2 $${I}_{k}={\int}_{{{{\uplambda }}}_{{{{{{\rm{min }}}}}}}}^{{{{\uplambda }}}_{{{{{{\rm{max }}}}}}}}F\left(\lambda \right)R\left({V}_{{bgk}},\lambda \right){{{{{\rm{d}}}}}}\lambda,\,k=1,\,2,\ldots,\,N$$

which can be transformed into a matrix **I**_*k*_ = **R**^T^[*V*_*bgk*_, *λ*]·*F*[*λ*] by discretized approximation. To verify the capability to reconstruct varied incident-light spectra, two types of modulated input light are irradiated and spectral responsivity values at 25 different V_bg_ are applied (i.e., *N* = 25).

For the first case, three monochromatic lasers around 1220, 1310, and 1410 nm modulated by monochromator are measured, respectively. From Fig. 5c, it can be seen that the reconstruction spectra for all the three monochromatic lasers with approximate resolution of ~20 nm are of excellent agreement with the actual situation, which demonstrates that the applied regression algorithm works well. For the second case, broadband incident light modulated by optical filters is measured. Simultaneously, scanning spectral imaging is demonstrated in this stage. The imaging target is the combination of the graphical logo “B” and abbreviations “BJUT” of Beijing University of Technology, as shown in Fig. 5a. For each spatial position (*X*, *Y*), spectral information can be reconstructed to obtain the final spectral data cube (*X*, *Y*, *λ*), according to the principle shown in Fig. 5b. Then, the practical scanning image under broadband light is displayed in Fig. 5d. Obviously, the shape and edge in the image are very clear, consistent with the target. Picking up the spectral information of position A, of which the spectrum is shown in Fig. 5e. It is in good agreement with the reference result by conventional commercial equipment. If false colors are endowed during the NIR wavelength range between 1300 to 1470 nm, spatial distribution at some selected monochromatic wavelengths would be able to be extracted as schematically shown in Fig. 5f.

**Fig. 6:**
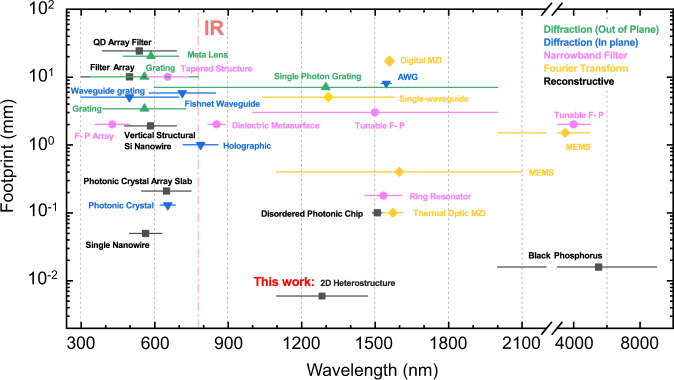
Plot of comparison between this work and other reported works for the key parameter footprint and spectral range. Five types of system are divided according to their operational mechanism^3^, including out of plane diffraction, in plane diffraction, narrowband filter, Fourier Transform, and reconstructive. The different colors and marks correspond to the operational mechanism. The line crossing the center marks represent the operational range. Different color indicates different technical routes. The triangle, inverse triangle, circle, diamond, and square marks represent the central response wavelength for each works.

Furthermore, a comparison of this 2D-vdWH spectrometer or spectral imager with some other typical miniaturized spectrometers for their footprints, operational spectral ranges, and mechanisms is summarized in Fig. 6 (see Supplementary Table 1 for more detail). Bounding free with those detector arrays or complex mechanical compositions, the demonstrated electrically tunable 2D heterojunction spectrometer has a distinct advantage in footprint with only 6 μm. In fact, it can be scaled-down to sub-micron further for this type of miniaturized spectrometer. Moreover, a designable energy band structure by different material combinations could easily extend the operational spectral range to IR wavelengths. Specially, a compact reconstructive spectrometer with a single black phosphorus photodetector was reported recently^44^, taking advantage of the tunable energy band of black phosphorus in the mid-infrared band. It further illustrates the possibilities for designing a micro-spectrometer available with an electrical tunable method. Although the example of the scheme here is a NIR 2D-vdWH spectrometer, it could be further extended to the MIR band by selecting other materials depending on the energy band engineering, such as HfS_2_/WS_2_^45^. Notably, the proposed strategy with Au atoms enhanced interlayer coupling and transition in this work possesses the potential for designing an IR spectrometer and spectral imager in terms of the material ambient stability.

## Discussion

In conclusion, we designed an electrically tunable 2D materials heterojunction-based near-infrared spectrometer and spectral imager. To demonstrate the spectroscopy function, a universal approach with heavy metallic atoms enhancing interlayer excited state transition dipole moment in 2D heterojunction is proposed. Following theoretical simulation prediction, experimental measurement confirmed that intercalating heavy metallic atoms can enhance the IEX photoresponsivity, enabling the efficient probing of an electrically tunable infrared photoresponse. Since our design does not have any integrated precise optical components or closely packed detector array structures, the footprint scales down to 6 μm. Considering the advantages of interlayer exciton absorption, i.e., designable interlayer transition energy by different materials combination and strong electrical tunability, the demonstration of this ultra-miniaturized near-infrared spectrometer shows an alternative way towards on-chip spectroscopy instruments over an extremely wide spectral range in future.

Simultaneously, we have to pay attention to photoresponsivity. The little wave function overlap between electrons and holes for interlayer exciton becomes the most critical limiting factor for light absorption, further the photoresponsivity. Although we have proposed an effective strategy by introducing metal intercalated vdWH to realize enhancement of optical absorption of interlayer exciton, it is still finite. More experimental efforts are required to solve this issue. First of all, more metal species such as Pt, Pd, etc. are valuable to be explored in order to achieve higher enhancement of the absorption in view of the latest developed metal single atom fabrication technique^46^. Next, enhancement of light matter interaction by the micro-nano optical component, e.g., plasmon, cavity or metasurface, is another potential way to increase optical absorption of interlayer exciton, which is inspired by the effective tuning of organic charge transfer (CT) state by optical microcavity coupling^47^. In addition, the construction of polar janus 2D material heterostructure with broken mirror symmetry, reduced layer spacing and an intrinsic vertical dipole moment may be potential to strongly enhance the interlayer coupling^48^. Moving forward, it is necessary to contribute more efforts to better reflect the advantages of optical absorption of interlayer exciton in future optoelectronic applications.

## Methods

### Device fabrication

Construction of the ReS_2_/Au/WSe_2_ heterojunction includes three steps. ML WSe_2_ sample is firstly exfoliated on the Si/SiO_2_ (285 nm) substrate by polydimethylsiloxane (PDMS) assisted dry transfer methods^49^. Then Au atoms are deposited on the WSe_2_ by a classical photocatalysis method^50,51^. A solid particle of HAuCl_4_·xH_2_O (Aladdin, 0.03 g) was dissolved in mixed solvent of 9.5 ml deionized water fixed with 0.5 ml methanol. The sample was immersed in the Au^3+^ solution and subject to ultraviolet (UV) light with 365 nm irradiation for 1 min, followed by a deionized water cleaning process and drying process at 80 °C for 6 h. The detailed chemical reaction process is below:

3 $${{{{{{\rm{WSe}}}}}}}_{2}+{{{{{\rm{hv}}}}}}\to {{{{{{\rm{WSe}}}}}}}_{2}+{{{{{\rm{e}}}}}}+{{{{{\rm{h}}}}}}$$

4 $${\left({{{{{{\rm{AuCl}}}}}}}_{4}\right)}^{-}+{{{{{\rm{e}}}}}}\to {{{{{{\rm{Au}}}}}}}^{0}+{{{{{{\rm{Cl}}}}}}}^{-}$$

After that, ReS_2_ ML is exfoliated and overlapped on the WSe_2_. To enhance the interlayer coupling, mild vacuum annealing at 250 °C for 5 h was processed. Electrical contacts Ti/Au (10 nm/70 nm) were fabricated by the UV lithography technology (SUSS MJB4) and e-beam evaporation (HHV FL400), followed by a lift-off process.

### Materials characterization

The morphology of heterojunction is characterized by optical microscopy (Olympus BX 51). To verify the molecular vibration modes and the optical band gaps of the applied 2D materials, a confocal Raman spectroscope (WItec alpha 300) is used to measure the Raman spectra and PL spectra at room temperature. To further characterize the thickness of the heterojunction, finer surface topography is performed by atomic force microscope (Bruker Multi-Mode 8). To prove the existence of Au atoms, scanning transmission electron microscope & energy dispersive X-ray spectroscope (Titan-G2) operated at 200 kV accelerating voltage is employed to observe the atoms arrangements and obtain the distribution of elements. To calculate the Fermi level of each material in heterojunction, surface potential of the components of heterojunction is tested by a Kelvin probe force microscope (Bruker Multi-Mode 8).

The absorbance was measured by Fourier-transform infrared (FTIR) spectrometer (Bruker Vertex 80v and Hyperion 2000 microscope). Quartz was used for the substrate. As is known, the absorbance for extra-thin film on transparent substrate is related to the differential reflectance spectra Δ*R/R* ^52^,

5 $$\frac{\Delta R}{R}=\frac{{R}_{{sam}}-{R}_{{sub}}}{{R}_{{sub}}}=\frac{4}{{n}_{s}^{2}-1}A$$

where *R*_*sam*_ and *R*_*sub*_ is reflection spectrum for the sample on the substrate and bare substrate, respectively. n_s_ is the reflective index of the substrate. A is absorbance of sample.

### Photoresponse characterization

The heterojunction device was encapsulated in the low-temperature chamber (Linkam, HFS600E-PB4) for the photoresponse characterization at 80 K. All the electrical and optoelectrical measurements were processed by a semiconductor parameter analyzer (Agilent B1500A). The light source for calibration of responsivities *R (V*_*bg*_*, λ)* is a supercontinuum light source (SuperK Extreme, NKT Photonics), of which the light is modulated by the monochromator (SOL Instruments, MS 2004i) and optical chopper. In addition, the power at each wavelength was equalized by an adjustable attenuator during the calibration.

### DFT calculation

The first principles calculations based on density functional theory (DFT) were performed on a 6 × 6 × 1 supercell. The calculations were implemented in Vienna Ab-initio Simulation Package (VASP)^53^ code with Perdew, Burke, and Ernzerhof (PBE) generalized gradient approximation (GGA)^54^ for exchange-correlation. Since the two layers of materials are combined through van der Waals force, the D3 method was employed for structure optimization, which was developed by Grimme et al^55^. The cut-off energy was set to 450 eV. The atomic structures were relaxed until the forces on each atom are below 10^−2 ^eV/Å and the convergence of total energy is lower than 10^−5 ^eV. The vacuum layer thickness is larger than 15 Å and the Brillouin zone was sampled by a set of 1 × 1 × 1 K-mesh. The transition dipole moment elements ${{{{{{\rm{M}}}}}}}_{{{{{{\rm{Z}}}}}}}$ and the unfolded band structures are obtained by Vaspkit^56^. The highest occupied energy levels in band structures were set as 0 eV.

### Electrical tunability calculation

Two-terminal model of the heterostructure device is built with 6.86 nm ML 2 × 2 WSe_2_−1 × 1 ML ReS_2_ as channel and the doped ML WSe_2_-ML ReS_2_ as electrodes. A vacuum space at least 16 Å is set perpendicular to the surface of the device to avoid the spurious interaction between periodic unit. According to our experiences in previous work, a 5 nm channel is enough to present CBM and VBM of 2D semiconductor FETs. The lengths of the left and right electrodes are semi-infinite. The device density of states (DDOS) is calculated by using DFT coupled with the non-equilibrium Green′s function (NEGF) method, which are implemented in the Quantum ATK 2020 package^57–59^. Transmission coefficient ${T}^{{k}_{\parallel }}\left(E\right)$ (${k}_{\parallel }$ is a reciprocal lattice vector point along a surface-parallel direction (orthogonal to the transmission direction) in the irreducible Brillouin zone (IBZ)) is calculated as

6 $${T}^{{k}_{\parallel }}(E)={{{{{\rm{Tr}}}}}}[{\varGamma }_{{{{{{\rm{L}}}}}}}^{{k}_{\parallel }}(E){G}^{{k}_{\parallel }}(E){\varGamma }_{{{{{{\rm{R}}}}}}}^{{k}_{\parallel }}(E){G}^{{k}_{\parallel }{{\dagger}} }(E)]$$

where, ${G}^{{k}_{\parallel }}$ is the retarded (advanced) Green′s function and ${\varGamma }_{{{{{{\rm{L}}}}}}/{{{{{\rm{R}}}}}}}^{{k}_{\parallel }}(E)={{{{{\rm{i}}}}}}({\sum }_{{{{{{\rm{L}}}}}}/{{{{{\rm{R}}}}}}}^{r,{k}_{\parallel }}-{\sum }_{{{{{{\rm{L}}}}}}/{{{{{\rm{R}}}}}}}^{a,{k}_{\parallel }})$ represents the level broadening due to the left electrodes and the right electrodes expressed in terms of the electrode self-energies ${\sum }_{{{{{{\rm{L}}}}}}/{{{{{\rm{R}}}}}}}^{{k}_{\parallel }}$, which reflects the influence of the electrodes on the scattering region. Doble- ζ Polarized (DZP) basis set is employed. The real-space mesh cutoff is of 520 eV, and the temperature is set at 300 K. The electronic structures of electrodes and central region are calculated with a Monkhorst–Pack^60^ 50 × 1 × 50 and 50 × 1 × 1 *k*-point grid, respectively. GGA of the PBE form to the exchange-correlation functional is applied in both the electronic structure calculations and the quantum transport simulations.

### Spectrum reconstruction

For an unknown incident spectrum *F*(*λ*), the corresponding photocurrent *I*_*k*_ in a fixed device is expressed as the followed integral from the available photodetectors array scheme^10,18^

7 $${I}_{k}={\int }_{{{{\uplambda }}}_{{{{{{\rm{min }}}}}}}}^{{{{\uplambda }}}_{{{{{{\rm{max }}}}}}}}F\left(\lambda \right)R\left(\lambda \right){{{{{\rm{d}}}}}}\lambda,\,k=1,\,2,\ldots,\,N$$

which can be transformed into a matrix **I**_k_ = **R**^T^[*λ*] × *F*[*λ*] by discretized approximation. In this work, a series of unique spectral photoresponse with different cut-off wavelength is achieved in a 2D heterojunction by electrical field instead of the complex design for a number of photodetector units. It means that all the detector units with different photoresponse spectral ranges are superimposed to a single unit by its electrically tunable feature. In another words, the signal probing shifts from space to time. When the incident light focuses on the active area of this device, sweep I_ph_-V_bg_ is processed to obtain the **I** = <*I*(*V*_*bg1*_), *I*(*V*_*bg2*_), …, *I*(*V*_*bgN*_)>. Then, the formula can be expressed as

8 $${I}_{k}={\int }_{{{{\uplambda }}}_{{{{{{\rm{min }}}}}}}}^{{{{\uplambda }}}_{{{{{{\rm{max }}}}}}}}F\left(\lambda \right)R\left({V}_{{bgk}},\lambda \right){{{{{\rm{d}}}}}}\lambda,\,k=1,\,2,\ldots,\,N$$

Finally, we utilized recursive least-squares algorithm and ‘ridge’ regression to complete reconstruction process.

### Spectral imaging

To achieve spectral imaging, home-building active imaging system was used, where a white light laser source is applied for illumination and our 2D heterojunction device is acting as detection element. The Nikon *Ni* optical microscope are used and act as beam splitter and mirror. All the optical accessories in this system have good NIR band applicability. The input illumination is reflected by the mirror and focused by the objective lens on the target surface. Then, the light can be reflected by the target. The 2D heterojunction device plays the role of “eye” to collect light information reflected by the target object through this optical system. In addition, an automatic stepping stage is applied for scanning imaging, which enables the detection for reflected light with different intensities or wavelength by the different position of the target object surface. The imaging target is the metal logo with shape of graphical “B” and “BJUT” based on quartz substrate. To ensure that the incident spectrum fits with the operational wavelength range of this device, optical filter (Thorlabs FEL 1300) was used. The spatial scanning steps at both X and Y axis are set as 1 μm considering the focused spot with diameter about 1 μm. With the termination of scanning process, initial photocurrent data cube (*X*, *Y*, *V*_*bg*_) can be established. In the initial data cube, each spatial position contains photocurrent values from each V_bg_. After reconstruction, spectrum information at fixed spatial position can be obtained, i.e., spectral imaging data cube (*X*, *Y*, *λ*). The reference spectrum at point A is measured by a commercial spectrometer (Princeton SpectraPro 500i).

## Supplementary information

Supplementary Information

## Data Availability

The data that support the findings of this study are available within the main text of this article and its Supplementary Information. Any other relevant data are available from the corresponding author upon reasonable request. Source data are provided with this paper.

## Source data

Source data

## Code availability

The algorithm code is available from the corresponding author upon request.
