# Supplementary material for: Electrically tunable two-dimensional heterojunctions for miniaturized near-infrared spectrometers
Source: Nat Commun. 2022 Aug 8;13:4627. doi: 10.1038/s41467-022-32306-z (PMC9360404; doi:10.1038/s41467-022-32306-z)
Supplement: Supplementary file 1 — Supplementary Information [file 41467_2022_32306_MOESM1_ESM.pdf]

Supplementary Information

Electrically Tunable Two-Dimensional Heterojunctions for Miniaturized Near-Infrared Spectrometers

Wenjie Deng[1,2,3,9], Zilong Zheng[2,9], Jingzhen Li[1,9], Rongkun Zhou[2], Xiaoqing Chen[1], Dehui Zhang[4,5], Yue Lu[6], Chongwu Wang[3], Congya You[1,2], Songyu Li[1,2], Ling Sun[2], Yi Wu[1,2], Xuhong Li[1,2], Boxing An[1,2], Zheng Liu[7], Qi jie Wang[3,8], Xiangfeng Duan[4,5,] * and Yongzhe Zhang[1,2,] *

[1]Key Laboratory of Optoelectronics Technology, Ministry of Education, Faculty of Information Technology, Beijing University of Technology, Beijing 100124, China

[2]Key Laboratory of Advanced Functional Materials, Ministry of Education, Faculty of Materials and Manufacturing, Beijing University of Technology, Beijing, 100124, China

[3] Centre for OptoElectronics and Biophotonics, School of Electrical and Electronic Engineering, Nanyang Technological University, Singapore 639798, Singapore

[4]Department of Chemistry and Biochemistry, University of California, Los Angeles, Los Angeles, CA 90095, USA

[5]California NanoSystems Institute, University of California, Los Angeles, Los Angeles, CA 90095, USA

[6]Beijing Key Laboratory of Microstructure and Properties of Solids, Faculty of Materials and Manufacturing, Beijing University of Technology, Beijing, 100124, China

[7]School of Materials Science and Engineering, Nanyang Technological University, Singapore 639798, Singapore

[8]Centre for Disruptive Photonic Technologies, School of Physical and Mathematical Sciences, Nanyang Technological University, Singapore 637371, Singapore

[9]These authors contributed equally: Wenjie Deng, Zilong Zheng and Jingzhen Li

*Corresponding Authors: yzzhang@bjut.edu.cn and xduan@chem.ucla.edu

Supplementary Figures

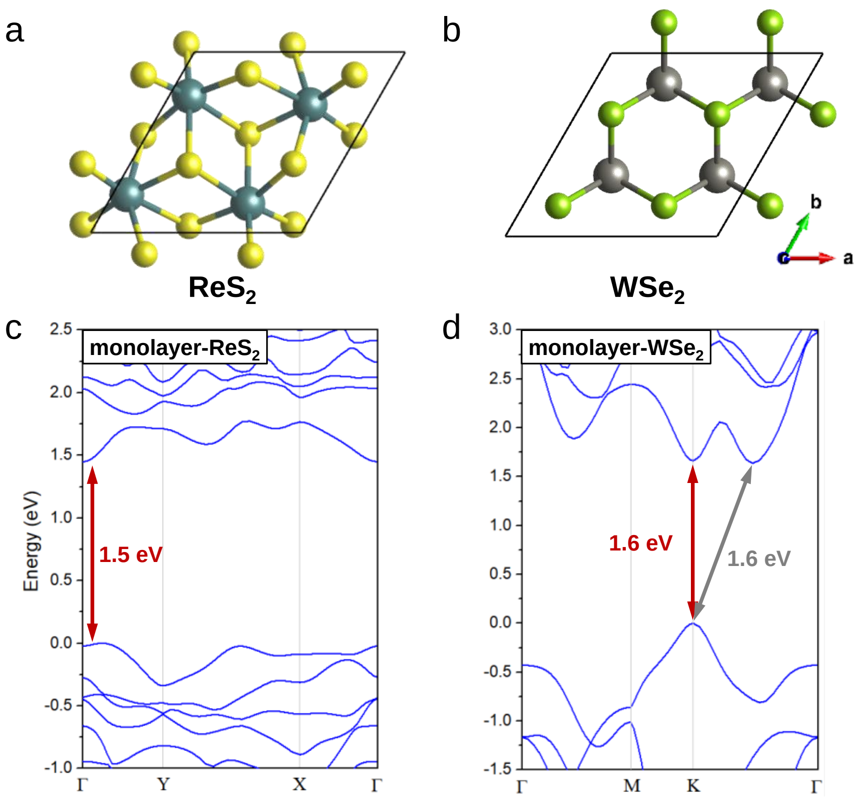

Supplementary Figure 1. The atomic geometries and the band structures of ReS_2 and WSe_2. (a) Top view of the atomic structure of monolayer ReS_2. (b) Top view of the atomic structure of monolayer WSe_2. (c) Calculated band structure of the monolayer ReS_2 with bandgap of 1.5 eV. (d) Calculated band structure of the monolayer WSe_2 with bandgap of 1.6 eV.

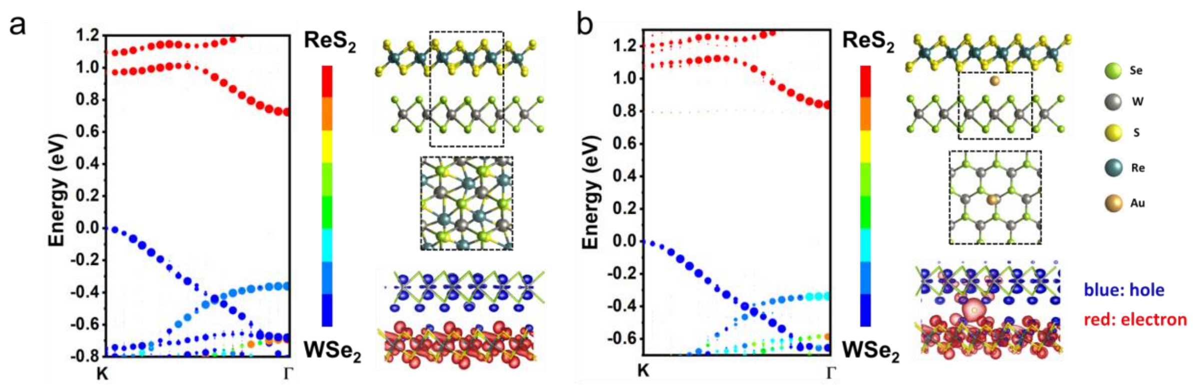

Supplementary Figure 2. The atomic geometries and the band structures of ReS_2/WSe_2 and $ReS_2/Au/WSe_2$. Atomic geometries, band structures, and electron distribution of CBM in WSe_2 layer (and hole distribution of VBM in ReS_2 layer) for twisted ReS_2/WSe_2 with 180° and (b) twisted $ReS_2/Au/WSe_2$ with 180°, respectively.

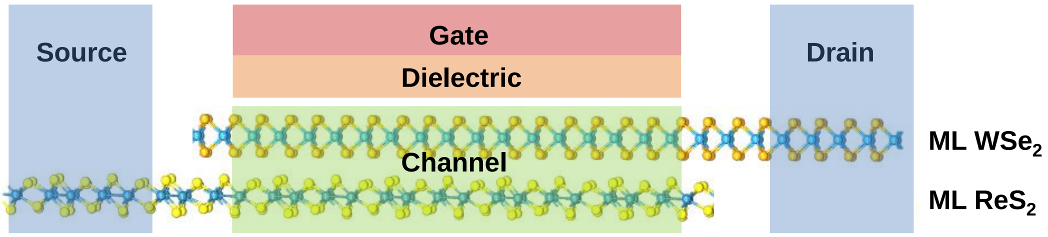

Supplementary Figure 3. Schematic diagram of a monolayer (ML) WSe_2-monolayer ReS_2 transistor. The transistor model includes a heterojunction channel with SiO_2 as dielectric layer. Source and drain electrodes are contact with ML ReS_2 and ML WSe_2 respectively.

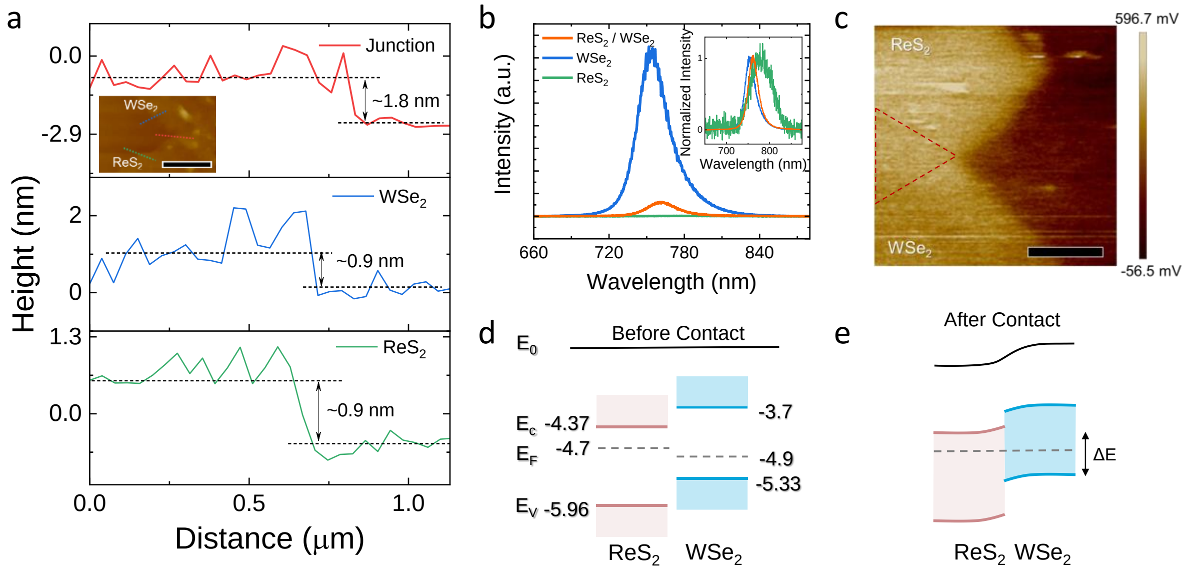

Supplementary Figure 4. Materials characterization of the 2D-vdWH. (a) The red, blue and green line are the height information across the dotted line in the inset atomic force microscope (AFM) image. Scale bar is 3 μm. They represent the junction area, WSe_2 layer and ReS_2 layer, respectively. both of these two layers are ~0.9 nm, which indicates that they are monolayers.[1,2] For the overlapping area, the height is ~1.8 nm, the sum of the thicknesses of the two monolayer materials. That also indicates that the vdW gap doesn't widen obviously by the metal intercalation under the current experimental conditions. The optical bandgaps and Fermi levels for ReS_2 and WSe_2 were probed by (b) room-temperature photoluminescence spectra (PL) and (c) Kelvin probe force microscopy (KPFM) test for device model building. Inset of (c): Normalized intensity results. The red dash line marks the heterojunction region. Clearly, the peaks at 1.59 and 1.64 eV indicated the optical bandgaps of monolayer ReS_2 and WSe_2, respectively.[2,3] The work function of ReS_2 and WSe_2 can be calculated by $eV_{cpd} = eV_{tip} - eV_{sample}$,[4] Where the V_{cpd}, V_{tip} and V_{sample}

are the contact potential difference, potential of the probe tip and surface potential of sample, respectively. Here, the tip potential is calibrated with ~5.2 V. Hence, the Fermi levels of ReS_2 and WSe_2 can be calculated as 4.7 and 4.9 eV. (d, e) Band energy diagrams of ReS_2 and WSe_2 before and after contact. E_0, E_C, E_F, E_V and ΔE are vacuum level, CBM, Fermi level, VBM and IEX energy, respectively. The ReS_2 layer is n-type with Fermi level in ~4.7 eV and the WSe_2 layer is p-type with Fermi level in ~4.9 eV.

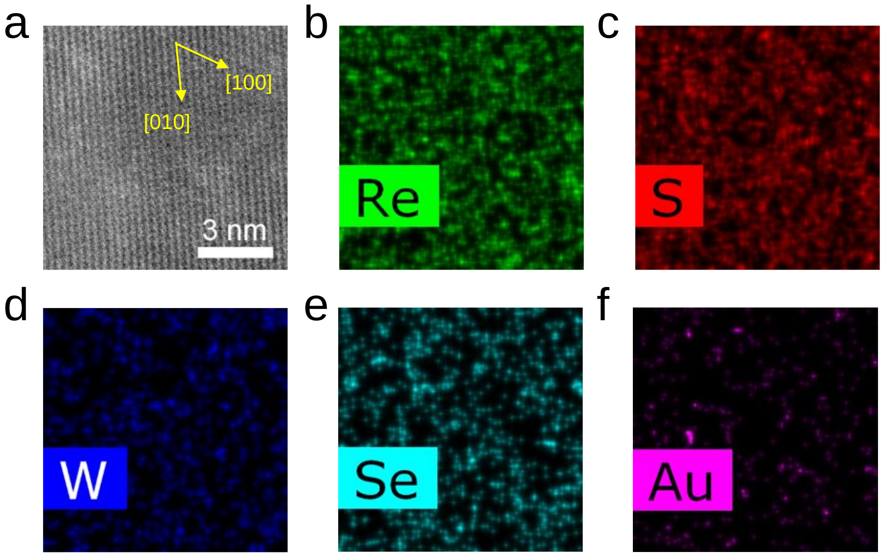

Supplementary Figure 5. Composition analysis by EDS mapping. (a) Top view TEM image of ReS_2/Au/WSe_2 sample, where the lattice orientation corresponding to the ReS_2 crystal structure. Corresponding elemental mappings of (b) Rhenium, (c) Sulfur, (d) Tungsten, (e) Selenium and (f) Gold. Energy dispersive X-ray spectroscopy mapping are performed for the ReS_2/Au/WSe_2 junction. The surface atomic arrangement in relative TEM image corresponds to ReS_2 with lattice orientation toward [100] and [010].[5] In the selected area, Re, S, W and Se were homogeneously distributed by the contribution of ReS_2 and WSe_2 layers. It is remarkable fact that the gold elements also exhibit sporadic distribution, revealing the successful fabrication of Au intercalated 2D heterojunction.

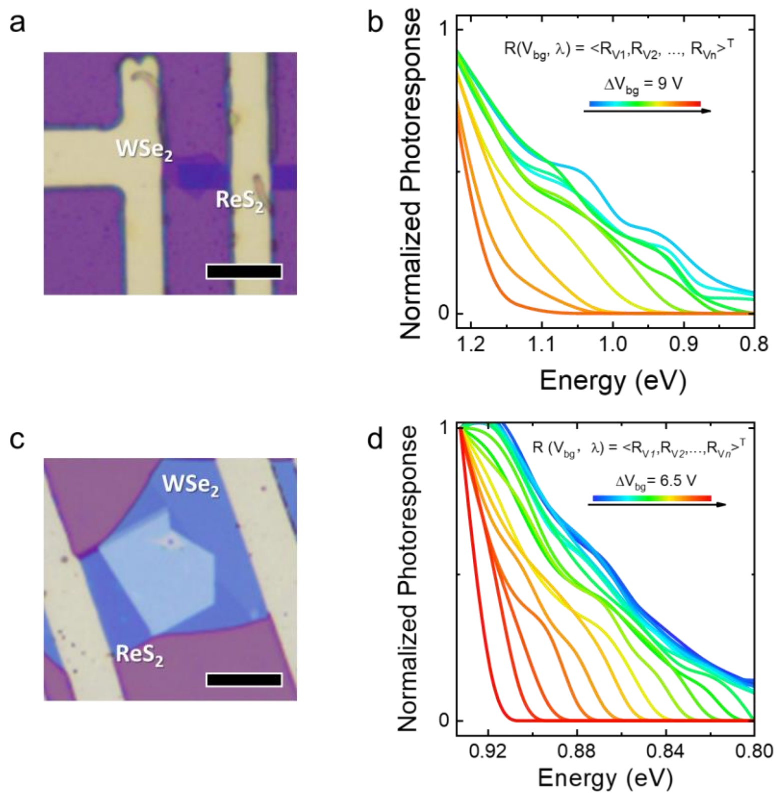

Supplementary Figure 6. Reproducibility of the 2D vdWH photodetector with electrical tunable band structure. (a) Optical image of the device 2# and its (b) Electrical tunable photoresponse. (c) Optical image of the device 3# and its (d) Electrical tunable photoresponse. Scale bar is 8 μm for (a) and (c). For device 2# when the gating voltage change about 9 V, the bandgap is tuned by ~0.2 eV. For device 3# when the gating voltage change about 6.5 V, the bandgap is tuned by ~0.12 eV The results exhibit similar trend compared with the device in the main text. Therefore, the electrical tunable devices applied for spectrometer are reproducible, even in a multilayer example. The multilayer example is characterized as shown in Supplementary Figure 7.

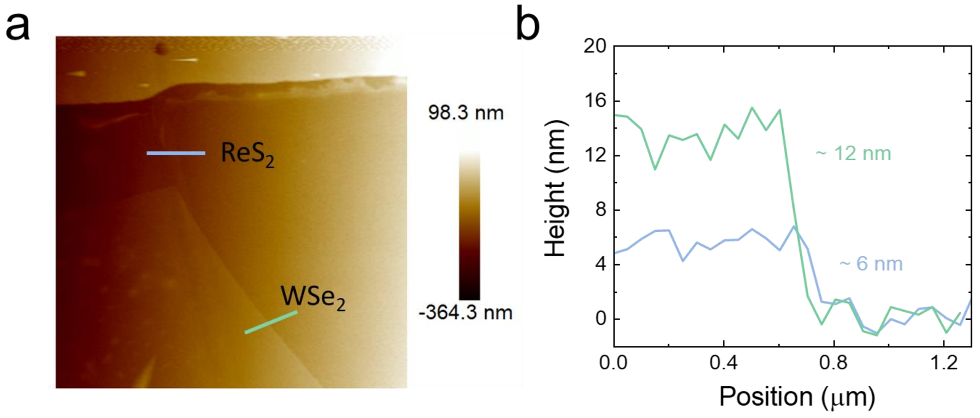

Supplementary Figure 7. Thickness of device 3#. (a) AFM mapping image of the device channel of 3# to extract the material thickness. (b) Linear profile along the blue and green lines for ReS_2 and WSe_2, respectively.

Supplementary Table 1. Comparison of different types of miniaturized spectrometer.

Principle	Wavelength range (nm)		Footprint (mm)	Peak Responsivity (mA/W)	Time (ms)	Spectral resolution (nm)	Ref.
Fabry–Perot etalons	360	500	2	12	0.01	18	6
Transmission grating	300	700	5	—	—	40	7
Transmittance-based filters	300	700	10	—	—	1	8
QD filter array	390	690	24	—	—	2	9
MEMS reflective concave blazed grating	340	780	10	—	—	12	10
Grating	390	730	3.4	—	—	3.4	11
Nanowire photodetectors array	500	630	0.05	10^7	3.5	7	12
Structurally Colored Silicon Nanowires	480	690	1.9	250	—	6	13
Meta-lenses	475	700	20	—	—	0.3	14
Photonic crystals slab filter	550	750	0.21	—	—	1.5	15
Linear Variable Optical Filter (LVOF)	570	740	10	—	—	0.7/2.2	16
Planar photonic crystal	652	660	0.13	—	—	1.2	17
Fishnet structure	580	850	5.75	88	—	16.5	18
Digital planar holograms	719	861	1	—	—	0.145	19
Dielectric metasurfaces	820	890	2	—	—	6	20
Two-dimensional heterostructure	1100	1470	0.006	0.01	20	20	This work
Echelle grating	600	2000	7	—	—	7	21
Single-waveguide FT spectrometer	1040	1580	3.2	—	—	5.5	22
Piezo-actuated Fabry-Perot Interferometers	1000	2000	3	—	0.2	1	23
Disordered photonic structure	1500	1520	0.1	—	—	0.75	24
Waveguide: microring resonators and microfluidic channels	1460	1610	0.18	—	—	1	25
Arrayed waveguide grating	1537	1557	8	—	—	0.2	26
Mach-Zehnder interferometer	1550	1570	17	—	—	0.1	27
Mach-Zehnder interferometer (MZI) cascaded with a tunable microring resonator	1532	1616	0.1	—	0.1	0.47	28
Double-cavity Fourier transform	1100	2100	0.4	—	—	7.5	29
Optical MEMS Translatory Actuator	2000	5000	1.5	—	0.2	135	30
Micromachined Fabry-Perot filter	3000	5000	2	—	—	86	31
Two-dimensional black phosphorus	2000	9000	0.016	—	—	90	32

Supplementary Reference

1. Yu, W., *et al.* Domain Engineering in ReS2 by Coupling Strain during Electrochemical Exfoliation. *Advanced Functional Materials* **30**, 2003057 (2020).
2. Tongay, S., *et al.* Defects activated photoluminescence in two-dimensional semiconductors: interplay between bound, charged and free excitons. *Scientific Reports* **3**, 2657 (2013).
3. Tongay, S., *et al.* Monolayer behaviour in bulk ReS2 due to electronic and vibrational decoupling. *Nature Communications* **5**, 3252 (2014).
4. Cheon, J.Y., *et al.* Intrinsic Relationship between Enhanced Oxygen Reduction Reaction Activity and Nanoscale Work Function of Doped Carbons. *Journal of the American Chemical Society* **136**, 8875-8878 (2014).
5. Cui, F., *et al.* Tellurium-Assisted Epitaxial Growth of Large-Area, Highly Crystalline ReS2 Atomic Layers on Mica Substrate. *Adv Mater* **28**, 5019-5024 (2016).
6. Correia, J.H., de Graaf, G., Kong, S.H., Bartek, M. & Wolffenbuttel, R.F. Single-chip CMOS optical microspectrometer. *Sensors and Actuators A: Physical* **82**, 191-197 (2000).
7. Dietmar, S., Duecker, M.O., Blume, O. & Joerg, M. Optical microspectrometer in SiON slab waveguides. in *Proc.SPIE*, Vol. 2686 (1996).
8. Oliver, J., Lee, W.-B. & Lee, H.-N. Filters with random transmittance for improving resolution in filter-array-based spectrometers. *Opt. Express* **21**, 3969-3989 (2013).
9. Bao, J. & Bawendi, M.G. A colloidal quantum dot spectrometer. *Nature* **523**, 67-70 (2015).
10. Finger-tip size, ultra-compact spectrometer head integrating MEMS and image sensor technologies. (www.hamamatsu.com/jp/en/product/type/C12666MA/index.html).
11. Kwa, T.A. & Wolffenbuttel, R.F. Integrated grating/detector array fabricated in silicon using micromachining techniques. *Sensors and Actuators A: Physical* **31**, 259-266 (1992).
12. Yang, Z., *et al.* Single-nanowire spectrometers. *Science* **365**, 1017-1020 (2019).
13. Meng, J., Cadusch, J.J. & Crozier, K.B. Detector-Only Spectrometer Based on Structurally Colored Silicon Nanowires and a Reconstruction Algorithm. *Nano Letters* **20**, 320-328 (2020).
14. Zhu, A.Y., *et al.* Ultra-compact visible chiral spectrometer with meta-lenses. *APL Photonics* **2**, 036103 (2017).
15. Wang, Z., *et al.* Single-shot on-chip spectral sensors based on photonic crystal slabs. *Nature Communications* **10**, 1020 (2019).
16. Emadi, A., Wu, H., de Graaf, G. & Wolffenbuttel, R. Design and implementation of a sub-nm resolution microspectrometer based on a Linear-Variable Optical Filter. *Opt. Express* **20**, 489-507 (2012).
17. Momeni, B., Hosseini, E.S. & Adibi, A. Planar photonic crystal microspectrometers in silicon-nitride for the visible range. *Opt. Express* **17**, 17060-17069 (2009).
18. Cadusch, J.J., Meng, J., Craig, B. & Crozier, K.B. Silicon microspectrometer chip based on nanostructured fishnet photodetectors with tailored responsivities and machine learning. *Optica* **6**, 1171-1177 (2019).
19. Koshelev, A., *et al.* Combination of a spectrometer-on-chip and an array of Young’s interferometers for laser spectrum monitoring. *Opt. Lett.* **39**, 5645-5648 (2014).
20. Yesilkoy, F., *et al.* Ultrasensitive hyperspectral imaging and biodetection enabled by dielectric metasurfaces. *Nature Photonics* **13**, 390-396 (2019).
21. Cheng, R., *et al.* Broadband on-chip single-photon spectrometer. *Nature Communications* **10**, 4104 (2019).
22. Pohl, D., *et al.* An integrated broadband spectrometer on thin-film lithium niobate. *Nature Photonics* **14**, 24-29 (2020).

23. Jarkko, A., *et al.* MEMS and piezo actuator-based Fabry-Perot interferometer technologies and applications at VTT. in *Proc.SPIE*, Vol. 7680 (2010).
24. Redding, B., Liew, S.F., Sarma, R. & Cao, H. On-Chip Random Spectrometer. in *Imaging and Applied Optics 2014* AM2A.4 (Optical Society of America, Seattle, Washington, 2014).
25. Nitkowski, A., Chen, L. & Lipson, M. Cavity-enhanced on-chip absorption spectroscopy using microring resonators. *Opt. Express* **16**, 11930-11936 (2008).
26. Cheben, P., *et al.* A high-resolution silicon-on-insulator arrayed waveguide grating microspectrometer with sub-micrometer aperture waveguides. *Opt. Express* **15**, 2299-2306 (2007).
27. Kita, D.M., *et al.* High-performance and scalable on-chip digital Fourier transform spectroscopy. *Nature Communications* **9**, 4405 (2018).
28. Zheng, S.N., *et al.* Microring resonator-assisted Fourier transform spectrometer with enhanced resolution and large bandwidth in single chip solution. *Nature Communications* **10**, 2349 (2019).
29. Eltagoury, Y.M., Sabry, Y.M. & Khalil, D.A. All-Silicon Double-Cavity Fourier-Transform Infrared Spectrometer On-Chip. *Advanced Materials Technologies* **4**, 1900441 (2019).
30. Thilo, S., Andreas, K., Christian, D., Harald, S. & Werner, S. Miniaturized FTIR-spectrometer based on an optical MEMS translatory actuator. in *Proc.SPIE*, Vol. 6466 (2007).
31. Norbert, N., Martin, E., Steffen, K. & Karla, H. Tunable infrared detector with integrated micromachined Fabry-Perot filter. *Journal of Micro/Nanolithography, MEMS, and MOEMS* **7**, 1-9 (2008).
32. Yuan, S., Naveh, D., Watanabe, K., Taniguchi, T. & Xia, F. A wavelength-scale black phosphorus spectrometer. *Nature Photonics* **15**, 601-607 (2021).
